# Profile of renal artery embolization (RAE) for renal trauma: A comparison of data from two major trauma center

**DOI:** 10.1590/S1677-5538.IBJU.2019.0506

**Published:** 2020-01-10

**Authors:** Jie Chen, Weicong Cai, Liping Li

**Affiliations:** 1 Injury Prevention Research Center Shantou University Medical College ShantouGuangdong China Injury Prevention Research Center, Shantou University Medical College, Shantou, Guangdong, P.R. China;; 2 Second Affiliated Hospital Shantou University Medical College ShantouGuangdong China Department of Urology, Second Affiliated Hospital of Shantou University Medical College, Shantou, Guangdong, P.R. China;; 3 Department of Non-communicable Disease Control and Prevention Shenzhen Center for Chronic Disease Control ShenzhenGuangdong China Department of Non-communicable Disease Control and Prevention, Shenzhen Center for Chronic Disease Control, Shenzhen, Guangdong, P.R. China

**Keywords:** Disease Management, Renal Artery, Embolization, Therapeutic

## Abstract

**Objective:**

To evaluate usage of renal artery embolization (RAE) for renal injuries and discuss the indications for this treatment.

**Materials and Methods:**

A retrospective study was performed evaluating the electronic medical records of all patients with renal trauma admitted to two major comprehensive hospitals in Shantou city from January 2006 to December 2015.

**Results:**

There were 264 and 304 renal traumatic patients admitted to hospital A and B, respectively. LGRT was the reason for presentation in the majority of patients (522, 91.9%). A total of 534 (94.0%) patients were treated conservatively. RAE was performed in 9 patients from 2012 to 2015 at hospital A, including in 6 patients (6/9, 66.7%) with LGRT, and 3 patients (3/9, 33.3%) with HGRT. No patient underwent interventional therapy (RAE) at hospital B during the same period. No significant differences in the operative rate of hospital A were observed between the two time periods (2006-2011 and 2012-2015). The operative rate for LGRT between the two hospitals from 2006 to 2011 and 2012 to 2015 was not significantly different. Hospital A showed a significant decrease in the rate of conservative treatment for patients with LGRT. In the univariate and multivariate analyses, the AAST renal grade both were significantly associated with undergoing RAE.

**Conclusions:**

LGRT was present in the majority of patients, and most cases of renal trauma could be treated with conservative treatment. RAE was well utilized for the treatment of renal trauma. However, some patients with LGRT were treated with unnecessary interventional therapy.

## INTRODUCTION

The kidney is the most frequently injured genitourinary organ in 1-5% of all trauma patients (1, 2). Approximately 245,000 cases of renal trauma occur worldwide per year (3), with an incidence ranging from 10.25 to 48.9 per million population (4, 5). Nearly three-quarters of patients with renal trauma are young and male (6). As the most common type of injury, blunt trauma remains a significant cause of morbidity and mortality in the population, accounting for 80-95% of renal trauma in Western countries caused by motor vehicle accidents, pedestrian accidents and falls (6-9), which is consistent with the findings of a study in China (10), although penetrating injuries are becoming more frequent due to an increase in violence, gunshot wounds and stabbing wounds. Given the well-protected anatomic location of the kidney, only major external forces lead to significant kidney injury.

Derived from clinical observations, the American Association for the Surgery of Trauma (AAST) kidney injury severity scale(Table-1) (11), which is based on computed tomography (CT) scan findings, classifies kidney injury into five grades, mainly including low-grade renal trauma(LGRT, I-III) and high-grade renal trauma (HGRT,IV-V); the AAST grade is the most important predictive variable of an increasing need for intervention (including vascular interventions and open surgery), and this tool is a useful practical instrument for clinicians to treat renal trauma properly.

**Table 1:** AAST renal injury grading scale (11).

Grade*	Description of injury
1	Contusion or nonexpanding subcapsular hematoma No laceration
2	Nonexpanding perirenal hematoma Cortical laceration < 1 cm deep without extravasation
3	Cortical laceration > 1 cm without urinary extravasation
4	Laceration: through corticomedullary junction into collecting system or Vascular: segmental renal artery or vein injury with contained hematoma, or partial vessel laceration, or vessel thrombosis
5	Laceration: shattered kidney or Vascular: renal pedicle or avulsion

*Advance one grade for bilateral injuries up to grade III.

With advances in imaging and treatment strategies, most cases of renal trauma can be managed conservatively to decrease the need for surgical intervention and increase organ preservation (12, 13). According to the guidelines of American Urological Association (AUA) (2) and European Association of Urology (EAU) (14) most patients with grade I-III renal trauma and even those with hemodynamically stable grade IV-V renal trauma can initially undergo conservative treatment (15-20). In particular, recent literature has provided more support for the application of conservative treatment in patients with HGRT to achieve good outcomes.

Renal artery embolization (RAE) was popularized in the 1970s by Almgard et al. (21). This technique has been increasingly applied to patients with renal trauma. According to the guidelines of the EAU (14), angiography or embolization can be used to diagnose and treat patients with stable hemodynamics, and good outcomes can be achieved, especially in patients with renal injury above grade III (15-17).

However, there is still no consensus on the selection principles for interventional therapy. The literature (22) has reported differences in the use of interventional therapy between interventional physicians and urologists, especially in patients with LGRT and some cases of overuse of interventional therapy. Furthermore, due to the inevitable risk and complications of interventional therapy, such as ectopic embolization and hemorrhage, it is of great significance to understand the appropriate application of and indications for interventional therapy in patients with renal trauma. This research will review the situation of renal trauma patients in two tertiary hospitals in the region in the past 10 years and elucidate the application and effects of interventional therapy for renal injury according to the different treatment methods of the two hospitals (one of which did not apply interventional therapy to treat renal trauma). We discuss the indications for RAE.

## MATERIALS AND METHODS

This study was approved by the 2^nd^ Affiliated Hospital of Shantou University Medical College Ethics Committee.

A retrospective study was performed on the electronic medical records of all patients with renal trauma collected from January 2006 to December 2015 in two hospitals (hospital A and hospital B) in Shantou city. The two hospitals are the major comprehensive hospitals affiliated with the medical university in Shantou city. These regional trauma centers have all medical capabilities, similar to the level I trauma centers described by the American College of Surgeons, Committee on Trauma (ACS-COT) (23). To provide standardization of the population, trauma patients were defined as any patient with an International Classification of Diseases, Tenth Revision, Clinical Modification code (ICD-10-CM) discharge diagnoses of S35.400-S35.402 and S37.001-S37.012. Patients with no renal injury (coding error) or iatrogenic renal trauma (as a complication) were excluded. In hospital A, the indication of treatment was obtained after consultation between urologists and interventionists, mainly according to the hemodynamic situations of the patients. If the hemodynamics was stable, yet the patients having progressive bleeding, such as progressive decrease of hemoglobin or progressive hematuria, IR treatment was given. If the patient had hemodynamic instability, renal exploration was performed. The rest of the stable patients were treated conservatively. In the hospital B, the indication of treatment was determined by the surgeons to determine whether to perform a renal exploration based on the hemodynamic condition. The treatment of renal trauma was categorized as renal-related operative management (RROM, including the following: nephrectomy (55.5), partial nephrectomy (55.4), renorrhaphy (55.81), nephrostomy tube placement (55.02-55.03), RAE or angioembolization (39.7928) and conservative treatment (including treatments in addition to the above two groups of renal injury patients). Conservative treatment was considered successful if renal-related surgery or RAE was avoided, even in patients who underwent surgery or interventional therapy that was unrelated to the kidney.

**Figure1:**
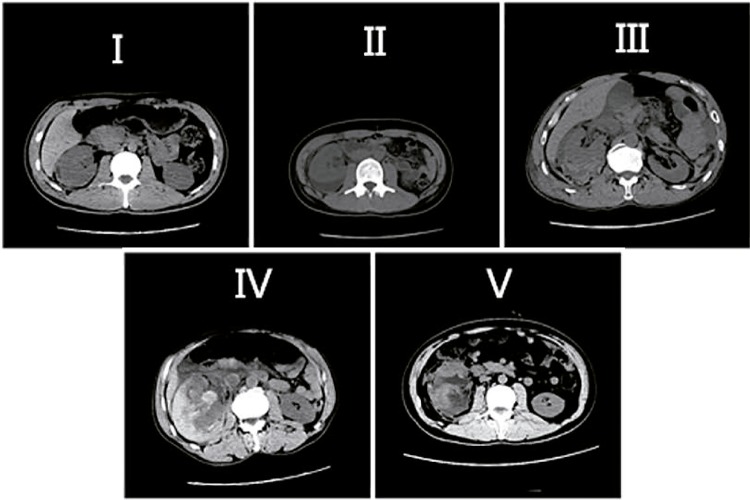
Renal trauma staging.

Data on gender, age, characteristics and mechanism of renal trauma, length of hospital stay (LOS), hematuria, clinical findings on presentation (blood pressure, heart rate), CT or ultrasonography findings, associated injuries, management and outcomes were recorded. Imaging for all patients was retrospectively reviewed by a radiologist experienced in renal trauma and graded using the AAST organ injury severity scale (Table-1) (Figure-1) (23).

Descriptive statistics were conducted to estimate the characteristics of all patients. Continuous data that were normally distributed (i.e., age) were expressed as the mean and standard deviation (SD), and those that were not normally distributed (i.e., LOS) were described using the median (interquartile range, IQR), and the between-group differences were assessed with independent-sample t tests. Categorical variables are presented as numbers and percentages and tested for differences using the Pearson χ^2^ test.

Hospital A began to perform RAE for renal trauma in January 2012, but hospital B had not applied RAE for renal trauma in the same period. We therefore performed the following statistical analyses: (1) between-group comparisons of the operative rate of RROM at hospital A between 2006-2011 and 2012 -2015 (Pearson χ^2^ test); (2) comparison of the conservative treatment rates for LGRT at hospitals A and B between 2006-2011 and 2012-2015 (Pearson χ^2^ test); and (3) relevant factors for interventional therapy (multivariate logistic regression analysis after the Pearson χ^2^ test and independent-samples t-test). The association of relevant factors with interventional therapy stratified by renal AAST injury grade was determined by logistic regression analyses, with low-grade as the reference category. Statistical analyses were performed using SPSS software package (SPSS 23.0for Windows; SPSS, Chicago, IL, USA).and statistical significance was determined at two-sided p<0.05.

## RESULTS

There were 264 and 304 renal trauma patients admitted to hospitals A and B, respectively, over the 10-year period. On average, approximately 26 and 30 patients with renal trauma presented per year to hospitals A and B, respectively. There was no statistically consistent change in the number of admissions per year over this study period (P>0.05).

Table-2 presents the outcome data for all patients with renal trauma treated at the two hospitals. Of the 434 men and 134 women included in the study, the mean age was 33.11 years. The mechanism of injury was blunt in 87.9% of patients and occurred secondary to the following three causes: falls (38.9%), road traffic-related injury (36.3%) and injury due to being hit by a blunt object (12.7%). A total of 69 (12.1%) patients presented with penetrating renal trauma. The above data were not significantly different between the two hospitals.

**Table 2:** Basic information of the renal trauma patients admitted to hospitals A and B during 2006-2015.

	Hospital A (N=264)	Hospital B (N=304)	p
Male(%)	200 (75.8)	234(77)	0.733
Age, mean(SD), year	33.59(17.13)	32.63(16.69)	0.499
Hematuria	207(78.4)	220(72.4)	0.096
**AAST, (%)**			**<0.001**
I-III	252(95.5)	270(88.8)	
IV-V	12(4.5)	34(11.2)	
LOS, median (IQR), day	20.5(14.00,34.00)	16.0(9.00,30.00)	<0.001
Cost, median (IQR), RMB	16483.7(7888.72,43857.72)	15950.9(8408.51,33042.57)	0.189
Associated lesions, (%)	159(60.2)	253(83.2)	<0.001
**Mechanism of injury, (%)**			**0.533**
Blunt	233(88.3)	263(86.5)	
Penetrating	31(11.7)	41(13.5)	

The mean LOS for renal trauma patients over the 10-year study period was 20.5 and 16.0 days at hospitals A and B, respectively. There was no consistent change in the mean LOS per year over this study period.

According to the AAST renal injury grading system, the patients were classified as grade I, II, III, IV and V renal injury in 245 (43.1%), 186 (32.7%), 91 (16.0%), 35 (6.2%), and 11 (1.9%) patients, respectively. The majority of the patients (72.5%) had associated injuries.

Of the 568 renal trauma patients, 534 (94.0%) were treated conservatively (including the patients who underwent surgery and interventional therapy that was unrelated to the kidney). No patient treated with observation underwent renal-related surgery or RAE. All patients with grade I trauma were managed conservatively. The renal-related surgery rate increased from 1% among patients with AAST grade II injuries to 7.7% in those with grade III injuries to 22.9% in those with grade IV injuries to 72.7% in those with V injuries. RAE was performed in 9 patients from 2012 to 2015 at hospital A, including 6 patients (6/9, 66.7%) with LGRT, and 3 patients (3/12, 33.3%) with HGRT. No patient underwent interventional therapy (RAE) at hospital B during the same period.

**Figure 2:**
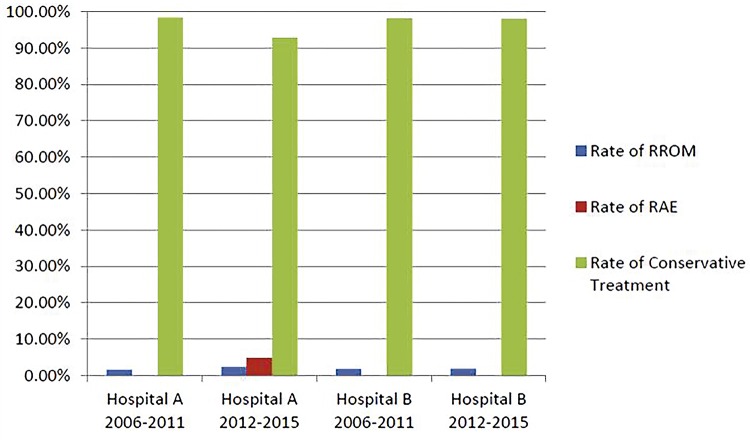
Comparison of the nonoperative rates for LGRT at hospitals A and B between 2006-2011 and 2012-2015.The conservative treatment rates of hospital A decreased from 98.41% to 92.86% ,with significantly different between two periods (p=0.008,χ2=9.761). The conservative treatment rates of hospital B were not significantly different between two periods (p=0.557, χ2=0.345).

As presented in Table-3, there were no significant differences in the operative rate at hospital A between the two time periods (2006-2011 and 2012-2015) (p<0.05). At the same time, the operative rate for LGRT between two hospitals in 2006 to 2011 and 2012 to 2015 was not significantly different (all p<0.05) (Figure-2). The operative rate for LGRT was not reduced due to the implementation of interventional therapy. However, hospital A showed a significant decrease in the rate of conservative treatment for patients with LGRT, which may be the reason for some patients undergoing interventional therapy.

**Table 3:** Comparison of the operative rates of patients with renal injury between 2006-2011 and 2012-2015 at hospital A.

	2006-2011 N=133, n(%)	2012-2015 N=131, n(%)	*p*	*χ*^2^
Treatment			0.683	0.22
Non-RROM*	131 (98.5)	128 (97.7)		
RROM	2(1.5)	3(2.3)		

*including RAE and NOM.

In the univariate analyses, the AAST renal grade was significantly associated with undergoing RAE. Additionally, in the multivariate analysis, only the AAST renal grade (odds ratio [OR], 13.56; 95% confidence interval [CI], 2.91-63.06) was significantly associated with undergoing RAE (Table-4).

**Table 4:** Univariate and Multivariate Logistic Regression Model for Predicting RAE.

Characteristics	Univariate OR(95% CI)	*p*	Multivariate OR(95% CI)	*p*
Age, y	0.98(0.94-1.02)	0.34	-	-
LOS, mean, d	1.00(1.00-1.01)	0.51	-	-
Sex	2.63(0.32-21.40)	0.37	-	-
Hematuria (yes/no)	2.25(0.28-18.38)	0.45	-	-
Cost	1.00(1.00-1.00)	0.23	-	-
Associated lesions (yes/no)	0.52(0.14-1.97)	0.33	-	-
**Mechanism of injury**			-	-
Blunt	1.00(ref.)		-	-
Penetrating	0.94(0.11-7.76)	0.95	-	-
Shock (yes/no)	0.54(0.06-4.59)	0.57	-	-
Tachycardia (yes/no)	2.00(0.52-7.64)	0.31	-	-
AAST renal grade				
LGRT	1.00(ref.)		1 (ref.)	
HGRT	13.677(2.94-13.58)	0.001	13.56(2.91-63.06)	0.578~2.215

## DISCUSSION

Conservative treatment is currently the standard of care for LGRT and is increasingly recommended for majority of patients with HGRT if they are hemodynamically stable. Our findings from this 10-year retrospective review including data from two major comprehensive hospitals show that approximately 94.0% of renal trauma patients are managed conservatively (including patients who underwent surgery and interventional therapy that was unrelated to the kidney). No one patient required conversion for further treatment.

Patients with LGRT had a higher success rate with conservative treatment (96.94%). This is consistent with the results of previous literature (15-20) as well as the guidelines (2, 14) on the choice of treatment for renal trauma. Furthermore, we found that patients with HGRT (27/46, 58.70%) also had good outcomes. However, all the patients who were successfully managed with conservative treatment had grade IV renal injury; one patient with grade V renal injury died due to multiple organ trauma, and the rest required surgical treatment (9 cases) or interventional treatment (1 case).

Interventional therapy as an effective treatment for renal trauma has been widely used. The application of interventional therapy enables the patient to avoid the risk of kidney resection and retain as much renal function as possible while reducing trauma to the patient. The EAU guidelines recommend that angioembolization has a central role in the nonoperative management of blunt renal trauma in hemodynamically stable patients (15-17). Currently, there are no validated criteria to identify patients who require angioembolization, and its use in renal trauma remains heterogeneous. Generally accepted CT findings indicating angioembolization are active extravasation of contrast, arteriovenous fistula and pseudoaneurysm (24). The presence of both active extravasation of contrast and a large hematoma (>25 mm depth) predict the need for angioembolization with good accuracy (24, 25). In fact, angioembolization has been utilized in the nonoperative management of patients with all grades of renal trauma; however, it is likely to be most beneficial in the setting of HGRT (AAST >3). According to our results, we found that RAE can be used as an effective treatment for renal trauma, even HGRT. Two patients with grade IV trauma and one patient with grade V trauma were treated with RAE and had good outcomes. The same findings were described by Hotaling (26), who believed that patients with HGRT could receive interventional therapy if they were hemodynamically stable and that good results could be obtained. However, close observation of vital signs should be ensured, and secondary embolization or surgical treatment should be performed in a timely manner if necessary. This is a significant change from the previous view that patients with HGRT are not suitable for interventional therapy.

However, we also compared the operative rate before and after the application of RAE to the treatment of patients with renal trauma at hospital A (p<0.05) and found no significant difference. The application of RAE did not significantly reduce the operative rate of patients with renal trauma at hospital A. In the patients who were treated with interventional therapy, 66.67% (6/9) were LGRT patients. We compared LGRT patients who underwent conservative treatment at the two hospitals during different periods. Hospital A utilized interventional therapy for some LGRT patients, so the conservative treatment rates decreased significantly (p=0.008, χ^2^=9.761). At the same time, hospital B maintained the original treatment, and the conservative treatment rates were not significantly different (p=0.557, χ^2^=0.345) (Figure-2). The above results suggest that RAE did not reduce the operative rate of patients with renal trauma, especially those with LGRT, and reduced the conservative treatment rate of patients with LGRT. Thus, some patients with LGRT may have unnecessarily undergone interventional therapy. According to the literature (22, 27), different diagnoses and treatment protocols may be used or different clinicians may have different understandings of the indications for interventional therapy based on their own experience, which may lead to unnecessary interventional therapy for some patients.

This result leads us to think about which are the relevant factors for RAE. We performed a statistical analysis of single and multiple factors related to RAE and found that the AAST grade was an independent factor associated with RAE for renal trauma. Multivariate analysis showed that only the AAST grade was associated with RAE for renal trauma. Previous literature and guidelines have highlighted the same perspective and suggested that HGRT (AAST >3) should be a relevant factor for RAE. Therefore, we prefer to recommend interventional treatment in patients with HGRT.

Although RAE is a minimally invasive technique with decreased trauma and improved repeatability, there are still inevitable complications that may impart significant morbidity to trauma patients. Complications of RAE, such as pain, infection, hemorrhage, ectopic embolization, pseudoaneurysm, arteriovenous fistula, and arterial dissection have been reported (28-30). Although complications were not found in our study, some patients still bear the risk of complications. Furthermore, the average hospitalization cost for interventional therapy patients and other patients was 61337.3 RMB and 37006.2 RMB, respectively. The average LOS for interventional therapy patients and other patients was 43.22 days and 31.42 days, respectively. Patients who underwent RAE incurred higher expenses and had a longer LOS than those who underwent other treatments, although there was no significant difference between them.

## CONCLUSIONS

The results of this study showed that LGRT accounted for the majority of patients, and most renal trauma patients could be successfully treated with conservative treatment. RAE is a minimally invasive technique that is well utilized for the treatment of renal trauma. Good outcomes can be achieved in HGRT patients, and HGRT is also recommended as an indication for RAE. However, some patients with LGRT who should receive conservative treatment were treated with unnecessary interventional therapy. This may lead to increased hospital costs and LOS.
